# Revolutionizing Molecular Design for Innovative Therapeutic Applications through Artificial Intelligence

**DOI:** 10.3390/molecules29194626

**Published:** 2024-09-29

**Authors:** Ahrum Son, Jongham Park, Woojin Kim, Yoonki Yoon, Sangwoon Lee, Yongho Park, Hyunsoo Kim

**Affiliations:** 1 Department of Molecular Medicine, Scripps Research, La Jolla, CA 92037, USA; ahson@scripps.edu; 2 Department of Bio-AI Convergence, Chungnam National University, 99 Daehak-ro, Yuseong-gu, Daejeon 34134, Republic of Korea; 975pjh@gmail.com (J.P.); woojin1544@gmail.com (W.K.); dbsrl0218@gmail.com (Y.Y.); sanguni088@gmail.com (S.L.); kmalrpkr13@gmail.com (Y.P.); 3 Department of Convergent Bioscience and Informatics, Chungnam National University, 99 Daehak-ro, Yuseong-gu, Daejeon 34134, Republic of Korea; 4 Protein AI Design Institute, Chungnam National University, 99 Daehak-ro, Yuseong-gu, Daejeon 34134, Republic of Korea; 5 SCICS, Prove beyond AI, 99 Daehak-ro, Yuseong-gu, Daejeon 34134, Republic of Korea

**Keywords:** computational biology, protein engineering, artificial intelligence, molecular design, de novo protein design, therapeutic proteins, synthetic biology

## Abstract

The field of computational protein engineering has been transformed by recent advancements in machine learning, artificial intelligence, and molecular modeling, enabling the design of proteins with unprecedented precision and functionality. Computational methods now play a crucial role in enhancing the stability, activity, and specificity of proteins for diverse applications in biotechnology and medicine. Techniques such as deep learning, reinforcement learning, and transfer learning have dramatically improved protein structure prediction, optimization of binding affinities, and enzyme design. These innovations have streamlined the process of protein engineering by allowing the rapid generation of targeted libraries, reducing experimental sampling, and enabling the rational design of proteins with tailored properties. Furthermore, the integration of computational approaches with high-throughput experimental techniques has facilitated the development of multifunctional proteins and novel therapeutics. However, challenges remain in bridging the gap between computational predictions and experimental validation and in addressing ethical concerns related to AI-driven protein design. This review provides a comprehensive overview of the current state and future directions of computational methods in protein engineering, emphasizing their transformative potential in creating next-generation biologics and advancing synthetic biology.

## 1. Introduction

In recent years, the subject of computational biology has experienced rapid and significant expansion, leading to a fundamental shift in how we comprehend and manipulate biological systems. The impact of computational approaches on protein engineering and molecular design is especially noticeable, as they have completely transformed the capacity to create and enhance proteins with new and unique capabilities. The incorporation of computational methodologies alongside conventional biological methods has created new opportunities for advancement in biotechnology, medicines, and related disciplines. This collaboration has resulted in improved and focused approaches for manipulating proteins, finding new drugs, and creating innovative biomolecules with improved capabilities.

Computational methods are becoming essential for customizing proteins for different biotechnological uses. Each year, a variety of tools and methodologies are being created and improved to keep up with the growing needs and difficulties of protein engineering [1]. The progress in machine learning and artificial intelligence has greatly improved the precision of protein structure predictions and the detection of functional regions, enabling more accurate manipulation of protein activities [2]. The use of computational approaches has greatly influenced the field of enzyme design. These approaches have allowed for the development of proteins that have enhanced catalytic efficiencies and new functionality [3]. For example, the utilization of machine learning models to forecast protein stability and interactions has simplified the design procedure, enabling the quick creation and manufacture of proteins without the limitations of living cells.

The combination of computational and experimental methods has expedited the design process by allowing the development of targeted libraries for laboratory evolution. This has resulted in a reduction in the extensive sequence space that requires sampling [4]. Platforms such as Mutexa demonstrate attempts to develop intelligent ecosystems that integrate fast computation with bioinformatics and quantum chemistry, making the process of identifying potential protein variants more efficient [5]. However, there are still obstacles to overcome in expanding the use of these technologies and making them available to a wider group of academics. This is crucial in order to fully utilize their potential in addressing global issues like sustainable development and healthcare [6].

Computational methods have gained significance in the field of drug development, thanks to recent progress in deep learning and artificial intelligence. These advancements have made it easier to quickly identify a wide range of powerful and specific ligands. These advancements have the capacity to make the drug discovery process more accessible to the general public, offering new possibilities for the efficient creation of safer and more efficient small-molecule medicines. The advancement of computational tools and their integration with experimental approaches is paving the way for remarkable innovation and application in protein design within the field of synthetic biology.

The continuous progress in computational biology is paving the way for a forthcoming period of protein engineering and molecular design, marked by enhanced accuracy, efficiency, and creativity. In order to overcome current hurdles and fully utilize the promise of biotechnology and pharmaceuticals, it is imperative to integrate computational and experimental approaches as the area continues to develop. This study seeks to present a thorough summary of the most recent developments in computational approaches used in protein engineering and molecular design. It emphasizes the significant influence of these technologies on the field.

## 2. Machine Learning and AI Applications in Protein Design

### 2.1. Deep Learning Approaches

#### 2.1.1. Convolutional Neural Networks for Structure Prediction

**Figure 1:**
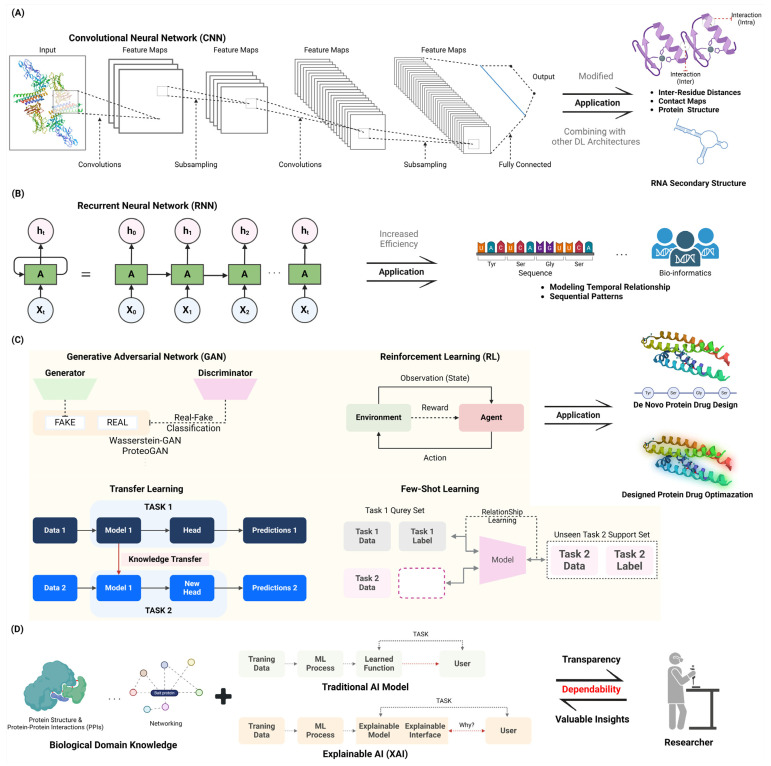
Development and application of AI algorithms in biotechnology. (**A**,**B**) Various AI algorithms significantly contribute to the development of biotechnology. Representatively, CNNs (Convolutional Neural Networks) are utilized for protein structure prediction through the prediction of distances and contact maps between residues. Additionally, RNNs (Recurrent Neural Networks) play a crucial role in sequence optimization through temporal relationship and sequential pattern modeling. (**C**) Recently, algorithms such as GAN (Generative Adversarial Network), RL (reinforcement learning), transfer learning, and few-shot learning have demonstrated their efficiency in modeling protein structures and interactions. These advanced algorithms are being utilized to overcome limitations in data collection required for model training, as well as limitations in designing new proteins. (**D**) Explainable AI (XAI) provides transparency and insight into modeling results by elucidating the decision-making process behind the vague “black box” judgment criteria of existing AI-based predictive models. Advances in AI algorithms have significant progressed protein engineering. However, they still require experimental validation. The integration of domain expertise and AI-based methodologies, also known as informed AI, can potentially enhance model efficiency and reliability and provide more accurate insights consistent with validated domain knowledge.

Convolutional Neural Networks (CNNs) are designed to automatically and adaptively learn spatial hierarchies of features from input data, making them particularly effective for tasks such as image classification, object detection, and semantic segmentation [7]. Recent advancements in CNN architectures, such as the development of attention mechanisms and the introduction of 3D CNNs for video analysis, have further expanded their capabilities and applications across diverse domains including medical image analysis, autonomous driving, and natural language processing [8]. CNNs have greatly enhanced the field of structure prediction in computational biology, specifically for proteins and RNA. CNNs are utilized for their capacity to do hierarchical feature extraction, rendering them well-suited for jobs that involve identifying intricate patterns in biological sequences and structures. CNNs have been utilized in protein structure prediction to forecast inter-residue distances and contact maps. This approach is exemplified in AlphaFold, which incorporates ResNets to improve prediction accuracy by incorporating translational invariance in the data [9,10]. In addition, CNNs have been modified for the purpose of predicting RNA secondary structure. Models such as CDPFold and E2Efold utilize convolutional layers to estimate the probability of base-pairing and then employ dynamic programming to extract the structure [11]. Recent progress has involved combining CNNs with other deep learning architectures, such as transformers, to enhance the accuracy of predicting protein secondary structures. This approach capitalizes on the benefits of both convolutional and attention mechanisms [12]. In addition, 3D CNNs have been used to forecast the local fitness landscapes of protein structures. This helps in recognizing the wild-type and consensus amino acids based on their structural contexts [13]. The applications mentioned highlight the flexibility and effectiveness of CNNs in solving various and intricate problems in structural bioinformatics. This makes them a fundamental component in the continuous development of computational biology [9,10,14] (Figure 1A).

#### 2.1.2. Recurrent Neural Networks for Sequence Optimization

Recurrent Neural Networks (RNNs) are a category of artificial neural networks engineered to handle sequential data by preserving an internal state or “memory” that enables information retention over time steps [15]. RNNs are a potent tool for optimizing sequences, demonstrating their effectiveness in modeling temporal relationships and sequential patterns. Recent developments in recurrent neural network topologies, including Long Short-Term Memory (LSTM) and Gated Recurrent Units (GRUs), have markedly enhanced their capacity to record long-term dependencies and alleviate the vanishing gradient issue [16]. Current studies have concentrated on enhancing RNNs for many purposes, such as predicting future values in time series data, understanding and generating human language, and analyzing biological information [17]. The convergence and performance of RNNs across many tasks have been greatly enhanced by the development of weight initialization schemes, such as Xavier/Glorot and He initialization [18]. Moreover, the utilization of optimization techniques such as adaptive learning rate approaches and gradient descent-based algorithms has played a vital role in improving the training efficiency and generalization performance of RNN models. Research has also investigated the combination of RNNs with other neural network structures, like CNNs, to utilize their complementary advantages for sequence modeling and feature extraction [19]. The adaptability and robustness of RNNs in sequence optimization are emphasized by these achievements, establishing them as essential components in the continuous progress of machine learning and artificial intelligence [17] (Figure 1B).

#### 2.1.3. Generative Adversarial Networks in De Novo Protein Design

In 2014, Ian Goodfellow and collaborators introduced a class of machine learning frameworks known as Generative Adversarial Networks (GANs). These frameworks are composed of two neural networks, a generator, and a discriminator that engage in a zero-sum game [20]. The generator’s objective is to generate synthetic data that can deceive the discriminator, while the discriminator endeavors to differentiate between genuine and fabricated data. This adversarial process enables GANs to acquire intricate data distributions and generate synthetic samples that are exceedingly realistic [21]. GANs have significantly transformed the field of de novo protein design by allowing the creation of new protein sequences that possess specific desirable characteristics. GANs including a generator and a discriminator network have demonstrated remarkable efficacy in modeling the intricate interactions between sequence, structure, and function that are inherent in proteins. Recent research has shown that GANs can be used to create proteins with specific structures and functions. This was achieved by using a Wasserstein-GAN with gradient penalty to design proteins with unique folds [22]. Furthermore, ProteoGAN, a conditional GAN, is intended to produce protein sequences by employing hierarchical functional labels that are derived from the Gene Ontology. This model outperformed other deep learning baselines in generating protein sequences [23]. The ability to produce proteins with precise enzymatic activity and solubility profiles has been improved by advancements in conditional generative modeling. This is exemplified by the hierarchical conditional GAN framework outlined. In addition, a comprehensive analysis was conducted on several deep generative models, emphasizing the crucial contribution of GANs in suggesting innovative proteins that closely mimic natural equivalents in terms of stability and expression [24]. The advancements highlight the profound capacity of GANs in creating new proteins with specific characteristics for various biotechnological and medicinal uses, demonstrating their ability to rapidly and effectively design proteins (Figure 1C).

### 2.2. Reinforcement Learning in Protein Engineering

#### 2.2.1. Optimization of Protein Properties

Reinforcement learning (RL) is a subfield of machine learning in which an agent acquires the ability to make decisions by interacting with an environment and receiving feedback in the form of rewards or penalties. The objective of RL is for the agent to acquire an optimal policy that maximizes cumulative rewards over time, without being explicitly instructed on which actions to take [25]. RL has demonstrated significant potential in the domain of protein engineering, namely in the enhancement of protein characteristics. RL techniques, like those used in ProteinRL, utilize generative protein language models to optimize protein sequences for specific structural and functional properties. This allows for the creation of new proteins with high charge content or diverse sequences that have high solubility and structural confidence [26]. Self-play RL is a new tool that helps optimize protein sequences to achieve desired features. This has a substantial impact on drug discovery and other biotechnological applications [27]. Moreover, the integration of RL with fitness landscape modeling, exemplified by the microFormer framework, enables the efficient exploration of the extensive mutant space. This integration facilitates the design of protein variants that exhibit improved activity and stability [28]. One recent development involves using protein language models as reward functions in RL frameworks to create biologically realistic sequences. These sequences are then optimized using smaller proxy models to efficiently handle computational expenses [27]. Model-based RL methods, like the ones that use AlphaZero, have shown success in protein backbone design. They outperform standard Monte Carlo tree search methods by adding secondary objectives and introducing new reward structures [29]. These discoveries demonstrate the profound impact of RL on protein engineering, enabling the development of proteins with customized characteristics for a wide range of uses in medicine, biotechnology, and synthetic biology.

#### 2.2.2. Design of Protein–Protein Interactions

RL has demonstrated considerable promise in the development of protein–protein interactions by facilitating the enhancement of binding affinities and the refinement of interaction specificities. Advancements in recent RL methods have resulted in the creation of advanced models capable of predicting and improving protein–protein interactions. An example of this is the RL pipeline that was created to find communities in weighted protein–protein interaction networks. This pipeline showed enhanced accuracy and speed in detecting new protein complexes, which emphasizes the scalability and efficiency of RL in this specific field [30]. Another significant contribution is the research that introduced the PPI-former model. This model utilized a large-scale dataset and SE(3)-equivariant representations to predict the effects of mutations on protein–protein interactions. The model achieved state-of-the-art performance in practical case studies, including SARS-CoV-2 antibody design [31]. In addition, the UniBind framework was introduced. It uses deep learning to examine protein–protein interactions at the residue and atom levels. This framework has been successful in accurately predicting the impact of mutations on binding affinities. Furthermore, it offers valuable insights into viral infectivity and variant evolution. This information is based on a study cited as [32]. These works highlight the significant influence of RL and deep learning in the field of protein engineering. This enables the creation of proteins with customized interaction features, which can be used in various fields such as medicine, biotechnology, and synthetic biology (Figure 1C).

### 2.3. Transfer Learning and Few-Shot Learning

#### 2.3.1. Leveraging Pre-Trained Models for Protein Design

Transfer learning is a technique in which the knowledge acquired from training a model on one task is applied to a related but distinct task. This method enhances the efficacy of tasks with restricted data by utilizing pre-trained models, which are frequently trained on extensive datasets. The exploration of cross-domain transfer learning and the development of more efficient fine-tuning techniques are among the most recent advancements in transfer learning [33]. Few-shot learning is a method that allows models to learn from a limited number of labeled examples, typically between one and five samples per class. This method is designed to resemble the learning process of a human, in which new concepts can be easily understood with minimal exposure. Meta-learning methodologies, metric learning, and data augmentation methodologies have been the primary focus of recent research in few-shot learning, with the objective of enhancing model generalization [34]. Transfer Learning and Few-Shot Learning are innovative methods in protein design that utilize pre-trained models to enhance protein properties with limited experimental data. These strategies facilitate the adjustment of models that have been trained on huge and varied datasets to specific protein engineering activities, thereby greatly minimizing the requirement for additional data gathering. For example, the effectiveness of pre-trained protein language models (PLMs) such as ESM-2 and ProGen in predicting protein fitness landscapes using few-shot learning was shown, thus improving the accuracy of protein design with little wet-lab data [35]. Furthermore, it was demonstrated how transfer learning may be utilized to optimize deep learning models for the purpose of predicting protein expression based on 5′UTR sequences in various situations. This approach enhances the ability of these models to generalize and be applied to varied genetic backgrounds [36]. A different significant work examined the combination of deep learning and transfer learning in protein design, emphasizing the potential of both techniques to create functional sites and develop new protein interactions with great accuracy [37]. The progress made in transfer learning and few-shot learning highlights the ability to transform protein engineering by facilitating the efficient and economical creation of proteins with specific properties for use in medicine, biotechnology, and synthetic biology (Figure 1C).

#### 2.3.2. Addressing the Challenge of Limited Data in Protein Engineering

The integration of powerful computational approaches and machine learning techniques has made it increasingly practical to tackle the obstacle of limited data in protein engineering. Efficient algorithms are necessary to navigate and optimize protein attributes due to the wide sequence space and combinatorial complexity of protein creation [38]. Machine learning models, namely those utilizing semi-supervised and transfer learning methods, have played a crucial role in estimating protein fitness landscapes with a small amount of experimental data. As a result, they have been able to guide protein engineering campaigns more efficiently [39]. In addition, data-driven methods have utilized high-throughput experimental data to enhance the catalytic activity and selectivity of enzymes, demonstrating the promise of machine learning in dealing with limited data availability [40]. By using a variety of training datasets, such as those obtained from X-ray crystallography, NMR, and cryo-EM, the performance of the model has been improved. This is achieved by reducing biases and enhancing the ability to apply the model to varied protein structures [41]. In addition, the utilization of evolutionary probability and stacking regression models has been employed to enhance protein characteristics, emphasizing the significance of computational techniques in addressing the constraints imposed by limited experimental data [42]. The progress made in computational and machine learning techniques highlights their crucial role in tackling the difficulties posed by limited data in protein engineering. This progress also paves the path for more effective and creative strategies for designing proteins.

### 2.4. Interpretable AI for Protein Design

#### 2.4.1. Explainable AI Models for Rational Protein Engineering

Interpretable AI, also known as Explainable Artificial Intelligence (XAI), is gaining recognition as an essential element in protein design. It provides transparency and valuable insights into the decision-making processes of machine learning models used for rational protein engineering. The incorporation of XAI techniques tackles the issue of the “black box” phenomenon that arises in intricate AI models, hence improving the credibility and dependability of forecasts [43]. For example, researchers have used feature attribution approaches and instance-based analysis to clarify the underlying mechanisms of protein–protein interactions. This has led to an improvement in the interpretability of prediction models [44]. The latest progress has shown the practical use of XAI in detecting DNA-binding proteins and enhancing the brightness of Green Fluorescent Proteins. This highlights the effectiveness of explainable models in real-world protein engineering activities. In addition, the advancement of self-explaining models and uncertainty assessment methods has made it easier to create proteins with specific features by offering clear justifications for model predictions [45]. These methods not only improve the clarity of the model but also provide guidance for experimental verification, guaranteeing that protein designs guided by AI are both dependable and efficient [46]. The integration of XAI into protein engineering pipelines is expected to transform the design and optimization of proteins, leading to more efficient and interpretable AI-driven solutions in biotechnology and synthetic biology [26] (Figure 1D).

#### 2.4.2. Integration of Domain Knowledge with AI-Driven Approaches

The fusion of domain expertise with AI-driven methodologies is an emerging field of study that seeks to improve the effectiveness, comprehensibility, and dependability of machine learning models. This approach, also known as informed AI, utilizes human experience to direct the development and improvement of AI systems, thus overcoming some limits that exist in solely data-driven methodologies. Embedding domain knowledge into AI models can greatly enhance their interpretability and resilience, as demonstrated by recent research in diverse domains like health, engineering, and environmental science [47]. Integrating clinical guidelines and expert knowledge into machine learning pipelines in the medical field has been proven to improve the accuracy, interpretability, and adherence to clinical standards of models, especially in situations where data are scarce [48]. Similarly, the utilization of many artificial intelligence agents that are specialized in different domains has shown to have greater capacities in discovering knowledge across other domains. This, in turn, enables the generation of more complete and precise insights. In addition, domain expertise can be included at different points in the AI pipeline, including data preprocessing, model training, and evaluation, to guarantee that the models are not only precise but also consistent with recognized principles particular to the domain [49,50]. This strategy, which combines data-driven and knowledge-driven techniques, tackles important difficulties such as expensive data collection and the risk of overfitting. As a result, it leads to the development of more generalizable and dependable AI systems [51]. Incorporating domain expertise is vital for the development of explainable AI systems, which are necessary for establishing confidence and enabling the ethical implementation of AI technologies in sensitive sectors such as healthcare and finance. In general, combining domain knowledge with AI-driven methods has great potential for enhancing the capabilities of AI systems, making them more efficient, dependable, and in line with human expertise and ethical standards [52].

## 3. Computational Methods in Enzyme Engineering

### 3.1. Structure-Based Design Strategies

#### 3.1.1. Homology Modeling and Threading Techniques

**Figure 2:**
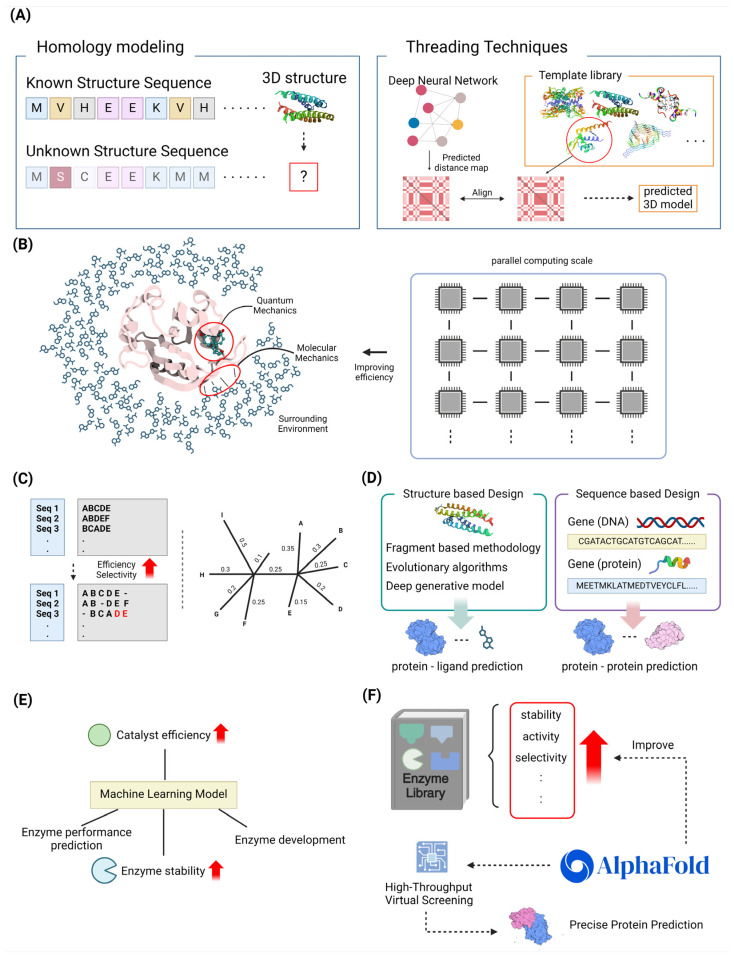
This figure illustrates the advanced computational techniques used in protein structure prediction, ligand–protein interaction modeling, and enzyme engineering. (**A**) Homology modeling (**left image**) infers the structure of a protein with an unknown structure by using the structure of a related sequence, based on the observation that proteins with similar sequences tend to have similar structures, while threading techniques (**right image**) predict a new structure by scoring the alignment of the target sequence against a template library with protein fold information when no structurally similar sequences are available; both methods are utilized for protein structure prediction in the absence of experimental data. (**B**) Quantum mechanics is used to predict the interactions between a ligand and a protein, while molecular mechanics is applied to model the interactions between a protein and its surrounding environment. The combined use of these two approaches, known as a hybrid method, has been enhanced by recent advancements in parallel computing technologies, overcoming previous limitations and contributing to the development of high-success-rate drugs. (**C**) The diagram on the left illustrates the process of aligning various protein sequences, enabling researchers to extract information more efficiently from refined sequences. Phylogenetic analysis allows for the determination of relative distances between elements, and by integrating MSA (Multiple Sequence Alignment) with phylogenetic approaches, information can be analyzed more effectively. (**D**) Structure-based design methods (**left**) are used for protein–ligand binding and provide examples of various underlying analytical techniques. Sequence-based design methods (**right**) are primarily applied to protein–protein interactions and can be broadly categorized into gene and protein sequence analysis. (**E**) Applying machine learning to enzyme engineering allows for predicting enzyme activity based on library data, improving enzyme stability, and facilitating enzyme development. It also helps explore methods to enhance the efficiency of catalysts or assists in selecting the appropriate catalyst. (**F**) The development of deep learning software such as AlphaFold3 has enabled rapid results in high-throughput virtual screening without the need for experimental procedures. Additionally, such software can significantly contribute to understanding enzyme–protein interactions within enzyme libraries, particularly in terms of stability, activity, and selectivity.

Homology modeling and threading are essential tools in structure-based protein design, enabling the prediction of protein structures in the absence of experimental data [53,54]. Homology modeling, also known as comparative modeling, is based on the assumption that proteins with comparable sequences would have similar structures. This makes it the preferred method when a homologous structure is present in the Protein Data Bank (PDB) [55]. This method has played a crucial role in the process of finding new therapeutics. It enables researchers to create accurate three-dimensional models of certain proteins, which helps them gain insights into how these proteins interact with drug molecules and aids in the development of novel medications Advancements in homology modeling, including superior sequence alignment methods and loop modeling techniques, have greatly improved the accuracy of these models, even for proteins that have a low sequence identity to their templates. Alternatively, threading, which is sometimes referred to as fold recognition, is used in cases where no homologous structures are present [56]. This method involves aligning the desired sequence with a database of established protein folds. A score system is then used to assess the compatibility between the sequence and each template structure [55,57]. Threading methods have advanced to include advanced algorithms, such as probabilistic graphical models and dynamic programming, in order to enhance alignment precision and model quality. Both techniques are essential components of contemporary drug discovery processes, facilitating the identification of potential targets for drug development and the creation of new therapeutic treatments using virtual screening and molecular docking. The combination of AI and machine learning has advanced these techniques, increasing their ability to forecast and operate efficiently. This integration also enables the management of extensive datasets produced by genomic and proteomic research [54]. In summary, the combination of homology modeling and threading approaches, supported by computational progress, remains a key driver of breakthroughs in predicting protein structures and designing drugs [53,55] (Figure 2A).

#### 3.1.2. Quantum Mechanics/Molecular Mechanics Approaches

Quantum mechanics/molecular mechanics (QM/MM) techniques have become indispensable in structure-based design methodologies, especially in drug development, because of their precise modeling of intricate biomolecular systems. Hybrid approaches integrate the accuracy of QM in modeling the active site with the efficiency of MM in representing the surrounding environment. This enables detailed simulations of enzyme reactions and interactions with ligands. Recent progress has been made in enhancing the scalability and efficiency of QM/MM simulations by utilizing exascale computing. This allows for the handling of huge biological systems and extended simulation timelines, which were previously difficult due to computational constraints [58,59]. The emergence of interfaces such as the MiMiC framework has showcased substantial parallel efficiency, facilitating the precise examination of thermodynamics and kinetics in drug targets with a high level of precision [58]. In addition, the use of machine learning techniques has increased the accuracy of QM/MM methodologies, making it easier to study energy transfer processes in biomolecular machines. The advancements discussed here demonstrate the potential of QM/MM techniques to significantly transform drug design. These approaches offer chemically precise insights into molecular interactions, leading to an enhanced success rate in drug development initiatives [60]. With the continuous expansion of computer resources, QM/MM approaches are in a position to make even more significant advancements in the field. These methods can tackle more intricate biological inquiries and facilitate more accurate therapeutic interventions [61,62] (Figure 2B).

### 3.2. Sequence-Based Design Methods

#### 3.2.1. Multiple Sequence Alignments and Phylogenetic Analysis

Multiple sequence alignment (MSA) and phylogenetic analysis are essential techniques for designing sequences based on their alignment and evolutionary relationships. These technologies have made substantial progress in recent years. The utilization of MSA is essential for a range of biological investigations, such as the estimation of phylogeny and the prediction of RNA structure. The scalability and accuracy of MSA algorithms, such as the EMMA (extending multiple alignments using MAFFT-add) technique, have been enhanced by recent advancements. These improvements are particularly beneficial for large datasets. The EMMA approach does this by efficiently managing computational resources through a divide-and-conquer strategy [63]. Researchers have also investigated bioinspired algorithms, which provide innovative methods to improve the precision and speed of alignment [64]. Phylogenetic analysis, which utilizes MSAs to deduce evolutionary connections, has been enhanced by advanced computer techniques such as maximum likelihood and Bayesian inference. These methods provide reliable frameworks for generating phylogenetic trees [65]. Recent research has shown that DNA sequences can be just as successful as protein sequences in determining deep phylogenies. This challenges long-held notions and broadens the range of phylogenetic approaches that can be used [66]. The integration of advanced computational tools and methods has supported these improvements, leading to better resolution and reliability of phylogenetic trees. As a result, our understanding of evolutionary processes has been enhanced [67]. As sequencing technology progress, it is crucial to continue developing and improving MSA and phylogenetic approaches. These advancements are essential for tackling intricate biological inquiries and pushing forward the discipline of bioinformatics [68] (Figure 2C).

#### 3.2.2. Coevolution-Based Approaches for Enzyme Design

Coevolution-based methodologies have become a potent instrument in the field of enzyme design. These methodologies utilize the evolutionary information included in protein sequences to pinpoint crucial interactions and mutations that can improve the activity of enzymes. These techniques employ numerous sequence alignments to identify coevolving residues, which are pairings of amino acids that have evolved together to preserve structural integrity and function. Notable progress in this area involves the creation of methods such as SCANEER (sequence co-evolutionary analysis to control the efficiency of enzyme reactions), which use sequence coevolution analysis to forecast enzyme performance. This enables the identification of specific mutations that can enhance enzyme efficiency and substrate selectivity [69]. These methods have effectively been used on several enzymes, such as beta-lactamase and aminoglycoside phosphotransferase, to show their ability to enhance enzyme activity for industrial and pharmacological purposes. In addition, the investigation of coevolution has played a key role in the identification of allosteric sites. These sites are essential for controlling enzyme activity and can be specifically targeted for the design of drugs [70]. The combination of computational tools and machine learning has increased the effectiveness of coevolution-based techniques, allowing for the creation of enzymes with new catalytic characteristics and enhanced stability [71,72]. As research progresses, coevolution-based methods are expected to have a crucial impact on the deliberate development of enzymes, providing valuable insights that connect natural evolution with synthetic biology.

### 3.3. Hybrid Methods

#### 3.3.1. Integration of Structure and Sequence Information

Hybrid approaches in drug and protein design combine both structure-based and sequence-based tactics to enhance the optimization of novel therapies. Structure-based design utilizes the three-dimensional structures of target proteins to uncover and enhance therapeutic candidates. This approach involves techniques such as fragment-based methodologies, evolutionary algorithms, and deep generative models, as demonstrated in recent works [73,74]. This method takes advantage of improvements in computational capacity and machine learning, which improve the ability to anticipate interactions between proteins and ligands and explore the field of chemistry [75]. Conversely, sequence-based design prioritizes the analysis of genetic and amino acid sequences in order to forecast protein activities and interactions. Direct coupling analysis and statistical modeling are employed to deduce co-evolutionary characteristics, which are essential for the advancement of hybrid proteins and genetic sensors [76,77]. By integrating the characteristics of both approaches, the integration of these methodologies in hybrid modeling provides a more thorough understanding of protein dynamics and function. This facilitates the design of more effective medications and proteins, as observed in the field of protein research [75]. Recent studies highlight the possibility of merging these tactics to overcome the inherent constraints of each method when employed separately, hence facilitating the development of inventive solutions in drug discovery and protein engineering [78] (Figure 2D).

#### 3.3.2. Machine Learning-Assisted Enzyme Engineering

Machine learning (ML)-assisted enzyme engineering is an advancing discipline that integrates computational and experimental methods to improve enzyme characteristics for many uses. Recent progress has shown that ML models can be used to forecast enzyme performance and stability, enhance catalytic efficiency, and assist in the logical development of enzymes. ML models can effectively explore the extensive protein sequence space to discover potential enzyme variations. This study focuses on the use of ML in predicting protein architectures and substrate specificity [79]. Moreover, the combination of ML with directed evolution has been demonstrated to expedite the process of enzyme optimization by lessening the workload of experiments. This highlights the significance of ML in providing guidance for directed evolution in the field of protein engineering [80]. In addition, the advancement of innovative machine learning algorithms, such as MODIFY (ML-optimized library design with improved fitness and diversity), has made it possible to simultaneously optimize both the effectiveness and variety of enzymes. This has greatly facilitated the identification of enzyme activities that are unique to the natural world [81]. The progress made in ML in enzyme engineering highlights the significant and profound influence it has, providing new opportunities for developing biocatalysts that have improved performance and unique capabilities (Figure 2E).

### 3.4. High-Throughput Virtual Screening

#### 3.4.1. In Silico Directed Evolution

High-throughput virtual screening (HTVS) and in silico directed evolution are innovative methods used in drug discovery and protein engineering. These methods utilize computing capacity to efficiently explore large chemical and protein spaces. HTVS employs computational models to efficiently assess extensive collections of compounds, discovering potential bioactive molecules without the necessity of physical synthesis. This approach overcomes the constraints of traditional high-throughput screening (HTS), which relies on pre-existing compounds [82,83]. Recent progress in machine learning, specifically Convolutional Neural Networks such as AtomNet, has shown great success in identifying new drug-like structures in different medical fields. This suggests that computational methods can effectively replace HTS in the early stages of drug discovery [83]. In silico directed evolution utilizes computational algorithms to model the process of evolution, enhancing protein functionalities through repeated cycles of mutation and selection. The utilization of deep learning models, such as AlphaFold2, has improved this method. These models are capable of accurately predicting protein structures, thereby enabling the creation of proteins with specific binding capabilities [84]. EvoPro is a new pipeline that combines deep learning to predict protein structure and optimize protein sequences. It demonstrates the effectiveness of in silico approaches in evolving protein binders. These computational methodologies not only speed up the process of discovery but also increase the range of chemicals and proteins that researchers may access, thereby enabling the development of unique therapeutic solutions [85,86] (Figure 2F).

#### 3.4.2. Computational Library Design for Enzyme Engineering

Computational library design for enzyme engineering is an innovative method that uses sophisticated computational techniques to enhance enzyme characteristics, including stability, activity, and substrate selectivity. This approach entails the generation of extensive and varied collections of enzyme variations, which can be computationally analyzed to pinpoint potential candidates possessing specific characteristics. The effectiveness of this technique has been greatly improved by recent breakthroughs in machine learning and structural bioinformatics. For example, advanced tools such as AlphaFold have brought about a significant transformation in the field of protein structure prediction. These tools enable researchers to precisely model enzyme structures and forecast the impact of mutations on enzyme activity [87,88]. Machine learning methods are being more and more utilized to analyze large datasets produced from high-throughput sequencing and screening. This allows for the detection of advantageous mutations and the forecasting of enzyme performance in different circumstances [89,90]. Computational approaches not only decrease the time and expense of traditional experimental methods but also broaden the range of enzyme engineering by exploring a wider sequence space. Computational library design is positioned to have a vital impact on the development of new biocatalysts for industrial and pharmacological purposes [3,87] (Figure 2F).

## 4. Molecular Dynamics Simulation Studies of Biomolecular Systems

### 4.1. Advanced Sampling Techniques

#### 4.1.1. Replica Exchange Molecular Dynamics

**Figure 3:**
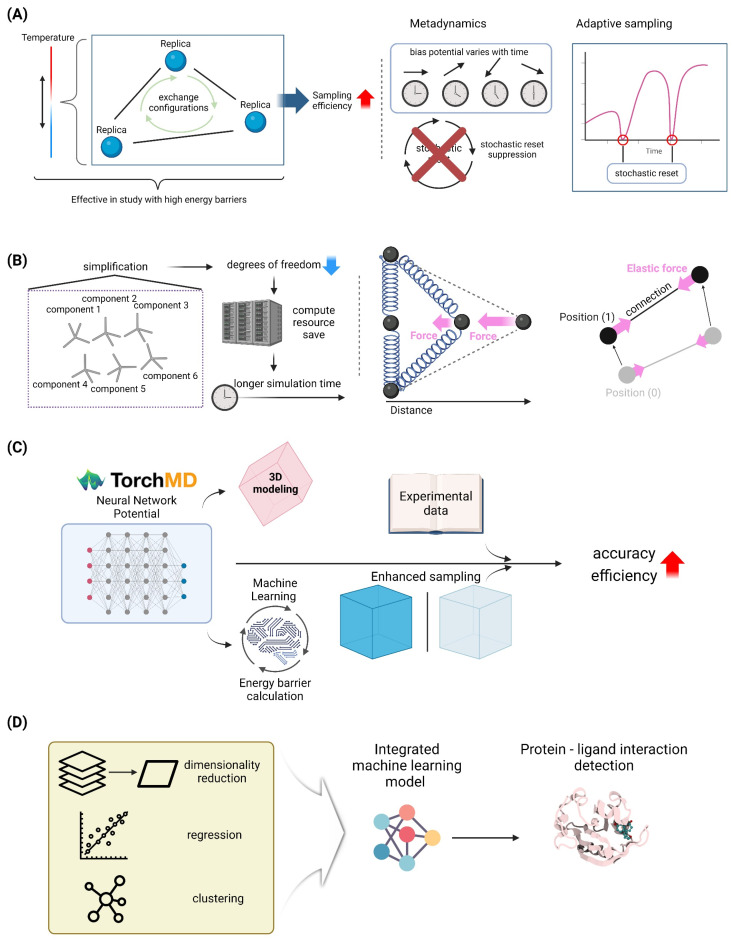
This figure illustrates various computational techniques used to enhance sampling efficiency and reduce computational resources in biomolecular simulations, highlighting their distinct approaches and applications. (**A**) Diagram of replica exchange molecular dynamics (**left**). This method forms multiple replicas and allows efficient simulation sampling through periodic exchanges of components between these replicas. It is particularly suitable for scenarios involving high-energy barriers in biomolecular interactions and can be conducted at different temperatures. Diagram illustrating the difference between metadynamics and adaptive sampling methods in terms of stochastic reset (**right**). Stochastic reset refers to the model probabilistically reverting to a previous state; metadynamics prevents this by introducing a bias potential, while adaptive sampling intentionally restarts the model at specific locations to enhance the sampling method. (**B**) Diagram of the MARTINI model and its advantages (**left**). The MARTINI model simplifies molecular systems by grouping multiple elements (primarily atoms) into larger entities called beads, rather than treating each element individually. This simplification reduces the degrees of freedom, significantly lowering computational resources required and enabling longer simulations with limited resources. Schematic of Elastic Network Models (ENMs) (**right**). ENMs represent the forces between biomolecules in large simulation environments using a spring model, where each node typically represents an alpha carbon. The longer the distance, the stronger the pulling force, allowing the possible conformations of biomolecules upon deformation to be inferred through this model. (**C**) Neural network potentials, such as Torch MD, enable 3D modeling and high-energy barrier calculations through machine learning. When combined with enhanced sampling techniques or experimental data, neural network potentials can achieve greater accuracy and efficiency. (**D**) An integrated model utilizing machine learning tools such as dimensionality reduction, regression, and clustering enables the modeling of complex biomolecular systems, such as detecting protein-ligand interactions.

Replica Exchange Molecular Dynamics (REMD) is a powerful enhanced sampling technique widely utilized in molecular dynamics simulations to overcome the limitations of traditional MD methods, particularly in exploring rugged energy landscapes of biomolecular systems. REMD involves simulating multiple copies, or replicas, of a system at different temperatures, allowing for the efficient sampling of conformational space by periodically exchanging configurations between replicas based on a Metropolis criterion. This method is particularly effective in studying systems with high energy barriers, such as protein folding, aggregation, and receptor–ligand interactions. Recent studies have demonstrated the utility of REMD in elucidating the mechanisms of protein aggregation associated with diseases like Alzheimer’s and Parkinson’s, as well as in the structural prediction of transmembrane proteins using implicit solvent models to reduce computational costs [91,92,93]. The method’s adaptability to parallel computing environments further enhances its efficiency, making it suitable for large-scale simulations on supercomputers [92]. Moreover, advancements such as the multicanonical replica-exchange method (MUCAREM) and the integration of implicit solvent models have been developed to improve sampling efficiency and reduce computational demands [92]. Overall, REMD continues to be a vital tool in biomolecular research, providing detailed insights into the dynamic behavior of complex systems at an atomic level (Figure 3A).

#### 4.1.2. Metadynamics and Adaptive Sampling Methods

Metadynamics and adaptive sampling approaches are essential tools in molecular dynamics (MD) simulations, specifically for investigating the intricate energy landscapes of biomolecular systems. Metadynamics improves the efficiency of sampling by introducing a bias potential that varies with time. This potential discourages the system from returning to states that have already been examined, enabling it to overcome energy barriers and explore novel conformations. The effectiveness of metadynamics relies heavily on the choice of collective variables, which must precisely reflect the sluggish phases of the system’s dynamics [94]. Recent advancements, such as the combination of stochastic resetting and metadynamics, have demonstrated potential in speeding up simulations even when less than ideal variables are utilized. This approach offers a substantial increase in speed without incurring any extra computing expenses [94]. However, adaptive sampling methods, such as adaptive path sampling and machine learning-enhanced sampling, maintain the thermodynamic ensemble while improving sampling by selectively restarting MD trajectories at specific locations. By employing deep learning, these techniques have proven to be highly successful in capturing protein conformational changes. They achieve this by accurately predicting the most favorable areas of the conformational space to investigate [95]. Ongoing research is dedicated to enhancing the efficiency and applicability of both metadynamics and adaptive sampling approaches. This study aims to broaden their scope to encompass a wider spectrum of biomolecular systems. By doing so, it will provide a more comprehensive understanding of protein dynamics and facilitate drug development efforts [95,96] (Figure 3A).

### 4.2. Coarse-Grained Models

#### 4.2.1. MARTINI Force Field and Its Applications

The MARTINI force field is a well-established coarse-grained model employed in molecular dynamics simulations for the investigation of biomolecular systems. It provides a favorable trade-off between computational efficiency and accuracy. The MARTINI model, created by Marrink et al., simplifies molecular structures by combining several atoms into larger “beads”. This simplification reduces the complexity of the system and enables simulations of massive biomolecular complexes over extended periods of time. This method has proven to be especially successful in replicating lipid membranes, protein folding, and interactions within intricate biological settings. The model MARTINI 3 has increased its application through recent advances. These advancements have improved the depiction of small molecules and increased the accuracy of lipid and protein simulations. This has been demonstrated in studies that have explored drug delivery systems and protein–protein interactions [97,98]. The integration of both top-down and bottom-up parameterization methodologies has enabled these improvements, resulting in a force field that accurately reproduces experimental partitioning free energies [99]. The MARTINI force field’s adaptability is emphasized by its successful integration into several simulation platforms, such as OpenMM, allowing for its extensive application in both academic and industrial research environments [100]. Continuing work in the field are focused on improving the model’s parameters and broadening its application range, namely in drug development and the examination of membrane proteins and cryptic pockets [98] (Figure 3B).

#### 4.2.2. Elastic Network Models for Large-Scale Simulations

Elastic Network Models (ENMs) are a widely used method in molecular dynamics simulations that are particularly useful for studying the overall movements of biomolecular systems. ENMs describe biomolecules as networks of nodes connected by springs, with the nodes commonly representing the Cα atoms of proteins. This representation enables the rapid calculation of normal modes and the study of slow, large-scale conformational changes. This approach is beneficial for investigating computationally challenging processes, such as protein folding, allosteric transitions, and massive biomolecular assemblies, which cannot be effectively studied using all-atom models. Recent progress has been made in improving the precision and usefulness of ENMs by combining them with other computational methods, such as molecular dynamics simulations and perturbation response scanning. This integration allows for the study of intricate systems, such as ubiquitin-specific protease 7 (USP7) and its mechanisms of allosteric regulation [101,102]. In addition, ENMs have been modified to different resolutions and parameterizations in order to accurately represent the dynamics of diverse biomolecular systems. This adaptation has shown resilience across numerous formalisms and applications [103]. These models are continuously improved to enhance their ability to make accurate predictions and to integrate them into multiscale modeling frameworks. This expansion increases their usefulness in the fields of structural biology and drug development [101,103] (Figure 3B).

### 4.3. Long-Timescale Simulations

#### 4.3.1. Specialized Hardware for MD Simulations

Advanced hardware has transformed long-term molecular dynamics (MD) simulations, allowing researchers to investigate biomolecular systems with exceptional precision and effectiveness. Notable progress has been made through the utilization of Graphics Processing Units (GPUs), Field-Programmable Gate Arrays (FPGAs), and Application-Specific Integrated Circuits (ASICs), each providing unique benefits in terms of velocity and computational capability. Originally intended for parallel processing in graphics, GPUs have been adapted to expedite MD simulations by effectively managing non-bonded interactions, resulting in a substantial decrease in computation time and cost [104,105]. FPGAs have the advantage of flexibility and efficiency, enabling the customization and optimization of MD algorithms. This customization can result in significant improvements in the speed of specific computational workloads [106,107]. ASICs, like the ones seen in Anton supercomputers, are designed exclusively for MD simulations. They provide impressive performance improvements by optimizing every component of the simulation process [105,108]. The hardware developments have increased the possible duration of simulations to the millisecond range and made MD simulations more accessible to a wider group of researchers. This has led to significant progress in drug discovery and structural biology [105]. The continuous advancement of technology is anticipated to boost the capabilities of MD simulations by integrating machine learning with specialized hardware. This integration will enable more detailed and precise examinations of complicated biomolecular processes.

#### 4.3.2. Enhanced Sampling Techniques for Accessing Biologically Relevant Timescales

Enhanced sampling approaches play a crucial role in expanding the time span of molecular dynamics (MD) simulations, allowing us to explore biologically significant time scales that would otherwise be impossible due to computational limitations. These methods, including metadynamics, replica-exchange molecular dynamics (REMD), and stochastic resetting, aim to tackle the difficulty of surpassing high-energy obstacles and investigating the complex energy patterns commonly found in biomolecular systems. Metadynamics is a method that improves sampling by introducing a bias potential that changes over time along specific collective variables. This helps to explore unusual events and calculate differences in free energy [94]. REMD, in contrast, utilizes the simulation of numerous duplicates of the system at various temperatures to enable effective sampling of diverse conformations by promoting transitions over energy barriers. Recent advancements, such as the integration of metadynamics with stochastic resetting, have shown substantial improvement in sampling efficiency. This improvement is observed even when suboptimal collective variables are employed, hence expanding the range of applications for these methods [94]. These advanced sampling techniques not only enhance the precision of MD simulations but also broaden their applicability in investigating intricate biological processes such as protein folding, ligand binding, and allosteric regulation. As a result, they contribute to the advancement of our comprehension of molecular mechanisms and assist in the discovery of new drugs [109] (Figure 3A).

### 4.4. Machine Learning-Enhanced MD Simulations

#### 4.4.1. Neural Network Potentials for Accurate and Efficient Simulations

Neural network potentials (NNPs) are a revolutionary method in molecular dynamics (MD) simulations that offer both precision and efficiency in modeling intricate biomolecular systems. NNPs utilize machine learning techniques to estimate potential energy surfaces, providing a computationally efficient alternative to conventional quantum mechanical calculations. This is especially advantageous for simulating extensive systems over extended durations. Recent technological developments, exemplified by TorchMD and its successor TorchMD-Net 2.0, have shown that NNPs may reliably simulate molecules that were not part of their training data. This demonstrates the ability of NNPs to generalize and perform well in diverse scenarios, indicating their robustness and versatility [110,111]. The models are trained utilizing data from accurate simulations or experimental observations, as demonstrated in the Differentiable Trajectory Reweighting approach. This method incorporates experimental data to improve NNPs without the need to differentiate through extensive MD simulations [112]. Moreover, incorporating active learning procedures, as explored in recent research, improves the capacity of NNPs to forecast infrequent occurrences, like bond breaking, by continuously updating the model with fresh data obtained through increased sampling approaches [113]. The inclusion of equivariance in neural networks, which acknowledges the spatial symmetries of molecular systems, has enhanced the precision and dependability of NNPs, rendering them a potent tool in both academic research and industrial applications [114]. These advancements highlight the capacity of NNPs to greatly enhance our comprehension of molecular dynamics, enabling major progress in fields like drug discovery and materials science (Figure 3C).

#### 4.4.2. AI-Driven Analysis of MD Trajectories

The utilization of artificial intelligence (AI) to analyze molecular dynamics (MD) trajectories has emerged as a revolutionary method for comprehending intricate biomolecular systems. This strategy harnesses machine learning (ML) to derive valuable insights from extensive datasets. By incorporating machine learning techniques, including as dimensionality reduction, clustering, regression, and classification, it becomes possible to analyze and interpret MD simulation data more efficiently. This overcomes the limitations of traditional methods that mainly rely on manual inspection and intuition [115]. Unsupervised deep learning techniques, such as graph neural networks, have shown promise in detecting complex patterns in MD data with many dimensions. They can capture the dynamics of protein–ligand interactions that are often difficult to analyze using traditional methods [116]. In addition, trajectory-based machine learning methods such as TrajML enable the development of precise force fields by training on ab initio molecular dynamics data. This improves the accuracy of MD simulations without the computational complexity associated with conventional techniques [117]. AI-enhanced techniques enhance the accuracy and efficiency of MD simulations and offer new opportunities to study protein dynamics, ligand-binding affinities, and other important biological processes. This ultimately contributes to the progress of drug discovery and materials science in fields such as [112,118]. The integration of AI with MD simulations is anticipated to better the modeling of intricate biomolecular systems, leading to greater understanding and allowing the development of innovative therapeutic approaches (Figure 3D).

## 5. Advances in Computational Docking and Drug Design

### 5.1. Protein–Ligand Docking

#### 5.1.1. Flexible Docking Algorithms

**Figure 4:**
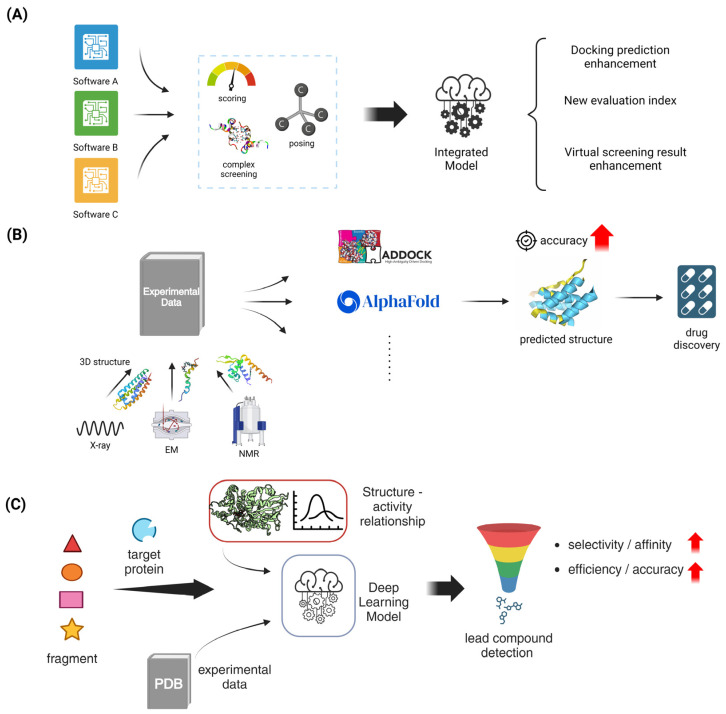
This figure highlights various approaches that enhance the accuracy and reliability of drug discovery processes by integrating computational models, experimental data, and deep learning methods. It showcases how combining these elements can improve prediction performance, structural accuracy, and lead compound optimization. (**A**) A model integrating output data from various software improves prediction performance, generates new evaluation metrics, and provides more reliable information during the virtual screening stage. Input parameters include docking scores, molecular (or component) poses, and representations of complexes. (**B**) Experimental data-based libraries enable the use of various software tools. These libraries compile 3D structures obtained through methods such as X-ray crystallography, electron microscopy, and NMR spectroscopy. By leveraging actual data, software like AlphaFold and HADDOCK can achieve highly accurate structural predictions, ultimately contributing to the drug development process. (**C**) A deep learning model for simulating the binding of lead compound candidates to target proteins can achieve superior performance by integrating structure-activity relationship data with experimental data. Experimental data can be sourced from databases like PDB, which mainly include data obtained from X-ray crystallography, electron microscopy, and NMR spectroscopy. Ultimately, the integrated deep learning model enhances selectivity and affinity during the lead compound optimization stage, improving efficiency and accuracy at every step.

Flexible docking methods have greatly improved the field of protein–ligand docking by enabling the dynamic modeling of ligands and protein targets. This has resulted in more accurate predictions of binding modes and has made drug development easier. Flexible docking is a docking method that allows for conformational changes in both the protein and ligand. This is important for accurately mimicking biological interactions, unlike typical rigid docking methods. Methods like as global optimization, step-by-step building, and multi-conformer docking have been created to investigate a broad spectrum of conformations, as observed in software applications like AutoDock Vina v1.2.5, DOCK v6.12, and Mdock v2.0. Although these methods require significant computer resources, they have demonstrated higher success rates in predicting the position of flexible ligands. However, they do not consistently beat rigid docking in virtual screening due to difficulties in accurately scoring the results [119]. Recent research highlights the importance of improved scoring methods that can precisely consider the energetic effects of ligand flexibility, including internal strain and changes in entropy [120,121]. Machine learning methods are getting more and more incorporated to improve the accuracy of scoring and decrease the computational expenses, which shows potential for breakthroughs in flexible docking approaches [120,122] (Figure 4A).

#### 5.1.2. Consensus Docking Approaches

The significance of consensus docking approaches in protein–ligand interactions has been emphasized by recent advancements in computer docking and drug design. These approaches have greatly enhanced the accuracy and dependability of predictions. Consensus docking approaches, which merge the outcomes of several docking programs, have been demonstrated to improve the results of virtual screening by averaging the scores or ranks of individual molecules. This approach overcomes the restrictions of using a single docking algorithm [123,124]. An example of this is the MetaDOCK method, which combines the data from Auto-Dock4.2, LeDock, and rDOCK. It has been shown to outperform individual programs in terms of scoring, posing, and screening protein–ligand complexes [125]. Furthermore, new consensus measures such as the Exponential Consensus Rank (ECR) have been created to overcome the drawbacks of conventional approaches. These metrics provide enhancements by employing rank-based techniques instead of score-based strategies, which are not influenced by score units and scales [123]. The integration of machine learning approaches enhances the prediction capacities of consensus docking, complementing these improvements. Consensus docking is anticipated to have a vital role in the rational development of therapies as the science advances. It will offer a thorough comprehension of molecular interactions and aid in the identification of new drugs [124] (Figure 4A).

### 5.2. Protein–Protein Docking

#### 5.2.1. Template-Based Docking Methods

Advancements in computational docking have greatly enhanced protein–protein docking techniques, with template-based docking emerging as a highly efficient method. Template-based docking utilizes the structural information obtained from known protein complexes to forecast the interaction surfaces of novel protein pairings. This method provides a more precise alternative to classic *ab initio* methods, but it requires the availability of suitable templates [126]. This method has been improved through the creation of extensive template libraries, such as those produced from the Protein Data Bank (PDB), which consist of several protein complexes that are used as benchmarks for docking predictions [127]. Recent research has shown that template-based approaches are useful in capturing the conformational dynamics of protein–protein interactions, which is crucial for accurately modeling these complexes. For instance, the combination of AlphaFold2 and template-based docking has demonstrated potential in accurately predicting protein complexes. This is achieved by employing deep learning algorithms to generate structural templates [128]. Furthermore, the utilization of paired interfacial residue restraints has been demonstrated to enhance docking predictions, particularly in situations requiring moderate to substantial conformational alterations [126]. With the continuous expansion of computer resources and structural databases, template-based docking is anticipated to have a growing significance in predicting protein–protein interactions. This will aid in advancing medication design and enhancing our comprehension of intricate biological processes (Figure 4B).

#### 5.2.2. Integration of Experimental Data in Docking Protocols

Computational docking has made substantial progress in improving protein–protein docking methods. This progress has been achieved by integrating experimental data, resulting in greater accuracy and dependability of docking predictions. Integrative methodologies that merge computational docking with experimental techniques, such as small-angle X-ray scattering (SAXS), electron microscopy (EM), and nuclear magnetic resonance (NMR), have demonstrated the ability to enhance docking success rates by offering supplementary structural constraints and filtering capabilities [129,130]. The integrative docking method, as reported by Trinh et al., employs simulated experimental data to enhance the accuracy of docking. This approach showcases the possibility of integrating different experimental methodologies to enhance the quality of docking models. In addition, techniques such as pyDockSAXS and HADDOCK have integrated SAXS data to improve and optimize docked models. This integration allows for better prediction of protein–protein interactions by utilizing low-resolution shape information [130]. By including evolutionary data, such as sequence conservation and coevolution, the accuracy of docking predictions is improved. This is achieved by gaining valuable information about the interface residues that are highly important for the interaction [130]. The incorporation of various experimental datasets into docking protocols is anticipated to have a significant impact on the advancement of the field. This integration, made possible by the continuous development of computational and experimental techniques, will enhance the accuracy of protein–protein interaction modeling and facilitate drug discovery endeavors (Figure 4B).

### 5.3. Fragment-Based Drug Design

#### 5.3.1. In Silico Fragment Growing and Linking Strategies

Advancements in fragment-based drug design (FBDD) have greatly improved the methods of in silico fragment growing and linking. These strategies are crucial in converting first fragment hits into powerful lead compounds. In silico methods, as reported by Moira et al., utilize computational tools to aid in the process of optimizing fragments into lead compounds. These methods integrate techniques such as hot spot analysis and structure–activity relationship (SAR) predictions to guide the expansion of fragments [131]. ACFIS 2.0 incorporates dynamic fragment growth techniques, which facilitate the comprehensive sampling of protein conformations. This enhances the precision of fragment binding predictions and enables the creation of a wide range of compound libraries [132]. Moreover, recent studies have emphasized the effectiveness of employing deep learning models in fragment optimization to expedite the discovery of synthesizable molecules. These models can predict bioactivity and pharmacokinetic features, thereby making the drug discovery process more efficient [131]. By combining computational tactics with experimental data from techniques like X-ray crystallography and NMR, the fragment growth and linking processes can be improved. This ensures that the final compounds have the best possible binding affinities and drug-like features [73]. With the increasing growth of computer power and algorithm sophistication, in silico tactics are anticipated to have a progressively vital part in the efficient development of new therapeutic medicines (Figure 4C).

#### 5.3.2. Machine Learning in Fragment-Based Approaches

We utilized machine learning techniques to augment the in silico fragment growing and linking tactics, resulting in a substantial improvement in the efficiency and accuracy of drug discovery operations. Recent studies in de novo drug design have demonstrated the successful application of machine learning models, namely those applying deep reinforcement learning (DRL), to optimize molecular structures. These algorithms learn how to change existing molecules in order to enhance their attributes [133]. By incorporating geometric deep learning frameworks such as fragment-based molecular expansion (FRAME), fragment-based drug discovery (FBDD) has been enhanced by properly determining the optimal locations for adding fragments to a ligand and assessing the geometric properties of these additions. This has resulted in improved predictions of the affinity and selectivity of the resultant molecules [134]. Moreover, the utilization of graph-based deep generative models in conjunction with evolutionary learning procedures has been utilized to optimize several objectives, including binding affinity and pharmacokinetic features, in the creation of innovative compounds [135]. These machine learning-based methods not only simplify the process of designing drugs based on fragments but also have the ability to efficiently explore large chemical regions, thereby enabling the rapid synthesis of new therapeutic agents. With the increasing computer power and advancement in algorithms, the incorporation of machine learning in FBDD is expected to have a significant impact on the future of drug discovery. This integration will allow for more accurate and efficient development of drug candidates.

### 5.4. Structure-Based Virtual Screening

#### 5.4.1. Pharmacophore Modeling and Shape-Based Screening

The merging of pharmacophore modeling with shape-based screening has greatly improved structure-based virtual screening, leading to substantial breakthroughs in the drug discovery process. Pharmacophore modeling is a technique that determines the specific arrangement of features required for molecules to interact with each other. It has been very useful in narrowing down large compound libraries to find potential matches. This has been demonstrated in several studies that have used databases like ZINCPharmer for efficient screening [136,137]. Shape-based screening enhances the analysis by emphasizing the compatibility of the ligand and the target protein in terms of their three-dimensional shapes. This approach has been improved with advanced algorithms like O-LAP, which enhances docking enrichment by comparing shape similarities with inverted binding cavities [138]. By utilizing these methods, it becomes possible to identify a wide range of compounds that have different structures but yet fulfill the requirements of pharmacophoric and form criteria. This enables the exploration of various molecular scaffolds and the finding of new potential drugs. Recent studies have emphasized the significance of machine learning in speeding up pharmacophore-based virtual screening. This allows for the effective management of large chemical spaces and enhances the identification of potential ligand candidates [139]. The advancement of computational tools and databases is likely to have a significant impact on drug design and development. The synergy between pharmacophore modeling and shape-based screening is anticipated to play a crucial part in this advancement [136,139] (Figure 4C).

#### 5.4.2. AI-Driven Virtual Screening Pipelines

The drug development process has been greatly improved by AI-driven virtual screening pipelines, which have transformed structure-based virtual screening. These advancements have led to increased efficiency and accuracy. AI-driven techniques utilize advanced algorithms to assess the intricate three-dimensional structures of target proteins and accurately forecast their interactions with prospective therapeutic molecules. This process greatly simplifies the discovery of highly promising candidates from extensive chemical libraries [140]. These technologies employ machine learning methods, namely graph neural networks (GNNs), to forecast chemical features and enhance drug design by properly simulating intricate molecular interactions [140]. AI has been successfully incorporated into virtual screening, resulting in faster drug discovery processes. One example is ZairaChem, a platform that utilizes AI/ML models to conduct quantitative structure-activity/property relationship modeling. This approach has significantly reduced attrition rates in experimental pipelines, as evidenced by research [141]. In addition, the use of AI-driven methods has allowed for the creation of prediction models that may estimate binding affinities without requiring substantial molecular docking. This has been demonstrated in studies where machine learning has expedited pharmacophore-based virtual screening [139]. These advancements not only expedite the quick detection of lead compounds but also make strong computational tools more accessible, thus enhancing the efficiency and cost-effectiveness of drug development efforts [6]. The incorporation of AI technologies into virtual screening pipelines is anticipated to boost the precision and speed of drug discovery, ultimately resulting in the development of safer and more effective treatments [142].

## 6. Design and Development of Novel Proteins with Enhanced Functionalities

### 6.1. De Novo Protein Design

#### 6.1.1. Computational Design of Protein Backbones

**Figure 5:**
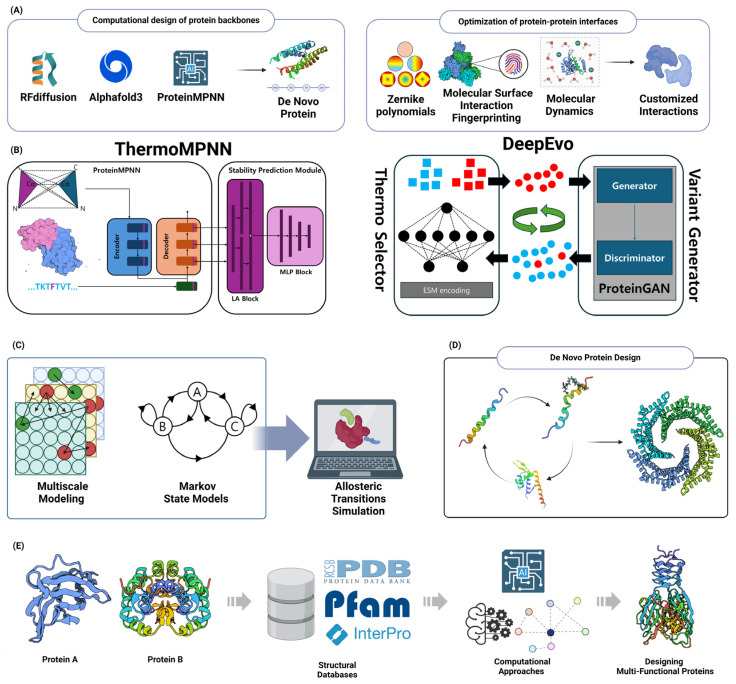
Enhanced functionalities of proteins through computational protein design and development. (**A**) Advancements in computational techniques, including deep learning models like RFdiffusion, AlphaFold2, and ProteinMPNN, have significantly improved de novo protein design. Zernike polynomials, Molecular Surface Interaction Fingerprinting (MaSIF), and molecular dynamics techniques help optimize protein–protein interactions. (**B**) ThermoMPNN is a computational tool that uses a deep neural network trained to predict stability changes in point mutations of a given protein with an initial structure. DeepEvo is an AI-based protein engineering strategy using a protein language model that can predict thermostability variants. (**C**) Allosteric transition simulations using multiscale modeling and Markov state models can predict protein functions, enabling the creation of customized allosteric regulatory proteins and the development of new protein functions. (**D**) Deep learning-based computational tools like Rosetta precisely modify protein structures to enhance binding capabilities, enabling the de novo protein design with customized binding properties. (**E**) Computational design for domain fusion and chimeric proteins uses structural databases and computer technologies such as machine learning to generate multifunctional proteins.

The field of de novo protein design has been greatly advanced by recent developments in computational techniques, namely in the design of protein backbones. These advancements have enabled the production of new proteins with improved capabilities. The advancement of complex algorithms, as described by MacDonald and Freemont, has enabled the integration of backbone plasticity into design processes. This overcomes the constraints of using rigid backbone templates and broadens the range of potential protein structures [143]. The ability to be flexible is extremely important for exploring a larger range of sequences and obtaining more intricate functionality. This has been emphasized by recent attempts to create new folds and functional sites using the extensive structural data found in the Protein Data Bank (PDB) [144]. RFdiffusion, an advanced technique, utilizes deep learning to generate novel protein backbones. This is achieved by repeatedly refining random residue frames. The results of this approach show substantial enhancements in the design of proteins with specific structural and functional needs [145]. In addition, the use of machine learning models, such as AlphaFold2 and ProteinMPNN, has significantly enhanced the effectiveness and achievement rates of de novo protein design. These models effectively forecast and optimize both the backbone structures and their related sequences, leading to improved efficiency [146]. These advancements not only improve our capacity to create proteins with specific functions but also open up possibilities for future use in biomedicine and synthetic biology, where precise manipulation of protein structure and function is crucial [144,147] (Figure 5A).

#### 6.1.2. Optimization of Protein–Protein Interfaces

Computational approaches have greatly improved the optimization of protein–protein interfaces through de novo protein design. These methods allow for exact engineering of molecular interactions, leading to greater functioning. Methods such as the use of Zernike polynomials have been created to represent the shape and electrical characteristics of binding sites. These methods enable the improvement of the compatibility of protein surfaces that interact with each other [148]. This method has effectively been used to create protein mutants that have stronger binding affinities. This has been proved in research that focused on the interaction between Ferritin and the Transferrin Receptor [148]. In addition, the incorporation of deep learning frameworks, such as Molecular Surface Interaction Fingerprinting (MaSIF), has introduced a new approach for capturing the essential geometric and chemical characteristics involved in protein–protein interactions. This method has greatly aided in the development of novel protein binders with high specificity and affinity [149]. The use of Monte Carlo simulations and molecular dynamics helps validate and improve interface designs, ensuring that altered proteins attain the expected functional outcomes [148]. As these approaches progress, they provide significant potential for use in synthetic biology and biomedicine. This is because they allow for the creation of proteins with customized interactions, which can lead to the development of new therapies and biomaterials [146,149] (Figure 5A).

### 6.2. Protein Stability Engineering

#### 6.2.1. Computational Prediction of Stabilizing Mutations

The latest developments in computational methods for predicting stabilizing mutations have greatly improved the field of protein stability engineering. However, the scarcity of these mutations still poses hurdles. ThermoMPNN, a type of computational tool, has demonstrated potential by obtaining a precision rate of 68% in predicting stabilizing mutations for proteins like the bacterial toxin CcdB. However, it has only shown small increases in thermal stability, with an increase of approximately 1 °C in the melting temperature [150]. Nevertheless, these methods frequently encounter difficulties when dealing with more intricate targets, such as influenza neuraminidase, underscoring the necessity for enhanced predictive precision [150]. Research has highlighted the drawbacks of existing techniques, pointing out that whereas several computational tools successfully forecast changes that cause destabilization, they struggle to reliably detect variants that promote stabilization [151]. Current endeavors have concentrated on amalgamating empirical data with computational forecasts to augment precision, as exemplified by logistic regression models that were trained on yeast surface display libraries. These models achieved a precision rate of 90% and a 3 °C elevation in thermal stability for CcdB [150]. In addition, RaSP, a type of deep learning model, has been created to quickly forecast changes in stability. This provides a scalable approach for analyzing protein variants on a wide scale. However, there are still difficulties in reliably predicting mutations that enhance stability [152]. The progress made in merging computational and experimental methods highlights the potential for improving the accuracy of predicting stabilizing mutations. This is essential for protein engineering and the creation of new proteins with improved functions [150,151,152] (Figure 5B).

#### 6.2.2. Design of Thermostable Proteins

Computational techniques have played a significant role in driving recent improvements in the design of thermostable proteins. These approaches have made it possible to engineer proteins with improved stability, which is beneficial for a range of industrial and biological uses. FireProt and its updated version, FireProt 2.0, are tools that have played a crucial role in automating the process of designing thermostable proteins. They achieve this by combining energy- and evolution-based methods to predict mutations that enhance stability. As a result, it becomes possible to create multiple-point mutants that exhibit improved thermal stability [153,154]. These platforms utilize both sequence and structural data, applying advanced algorithms to reduce antagonistic effects caused by mutations and improve stability without compromising function [153,154]. In addition, the utilization of deep learning models, such as DeepEvo, has made it possible to forecast thermostable variations by simulating evolutionary processes. This offers a new method for protein engineering that avoids the time-consuming old techniques. Molecular dynamics simulations have been important in comprehending the stability and dynamics of engineered proteins, providing valuable knowledge about the structural foundation of thermostability and driving the improvement of protein interfaces to promote functionality [155]. In addition, ancestral sequence reconstruction has become a promising approach that utilizes phylogenetic analysis to revive ancient proteins with naturally stable structures. This expands the range of tools that may be used to build strong proteins for commercial and medicinal purposes [156,157]. These computational advancements enhance the effectiveness of designing proteins that can withstand high temperatures and also create opportunities for their use in demanding conditions, thus progressing the area of protein engineering (Figure 5B).

### 6.3. Protein Functionalization

#### 6.3.1. Computational Design of Allosteric Regulation

The latest progress in the computational design of allosteric regulation has greatly improved the capacity to manipulate proteins and create new functions. This research has specifically concentrated on optimizing allosteric sites to achieve precise control over protein activity. The utilization of computational tools, as described by Duan et al., has played a crucial role in understanding the routes of allosteric communication. These methods have allowed for the identification and creation of allosteric sites that can be specifically targeted for the purpose of discovering new drugs [158]. These approaches employ bioinformatics and machine learning to simulate the dynamic and network-based characteristics of allosteric control. They offer valuable insights into the structural alterations that enable allosteric signaling [159,160]. Recent research has utilized multiscale modeling and Markov state models to simulate allosteric transitions. This approach provides a quantitative framework for predicting how mutations or ligand binding can affect protein function [159]. The combination of computational and experimental methods has improved these models, enabling the creation of proteins with improved allosteric properties. This has been demonstrated through the manipulation of allosteric networks to enhance enzyme activity and biosensor performance [161]. As these computational tools progress, they offer the potential to enhance the range of methods for creating proteins with customized allosteric regulation. This, in turn, will contribute to the advancement of synthetic biology and therapeutic development (Figure 5C).

#### 6.3.2. Engineering Proteins with Novel Binding Properties

The development of proteins with new binding properties has been greatly influenced by the use of computational and experimental methods to improve the specificity and strength of protein interactions. Computational tools like Rosetta have played a crucial role in the development of proteins with novel binding sites. These tools enable precise modifications to protein structure, resulting in improved binding capacities. Recent research on de novo protein design have emphasized the significance of these advancements [3,144]. These technologies employ algorithms that forecast the most effective interactions between proteins and their targets, enabling the development of proteins with customized binding properties for particular applications, such as therapeutic targets or biosensors [162]. Furthermore, machine learning techniques have been included in protein design in order to forecast and enhance binding interactions. This is achieved by utilizing extensive datasets from the Protein Data Bank to guide design choices and enhance precision [144]. Directed evolution is an experimental technique that complements computational methods. It involves iteratively refining protein sequences to acquire specific binding qualities. This process enhances the functionalization of proteins for various biomedical purposes [162]. The integration of these computational and experimental methods not only speeds up the progress of proteins with unique binding characteristics but also broadens their potential for use in areas such as pharmaceutical development and synthetic biology [162]. As these approaches progress, they provide the potential to improve the accuracy and effectiveness of protein engineering, leading to new and creative solutions in the fields of health and biotechnology [144,163] (Figure 5D).

### 6.4. Designing Multi-Functional Proteins

#### 6.4.1. Computational Approaches for Domain Fusion

Advancements in computational methodologies for domain fusion have greatly improved the design and creation of multi-functional proteins with new binding characteristics and capabilities. The fusion of protein domains enables the formation of chimeric proteins possessing distinctive combinations of functionalities. This process largely depends on precise predictions of both structure and function, as demonstrated in recent research utilizing AlphaFold II and other modeling techniques [164]. Computational approaches encounter difficulties in accurately anticipating the spatial orientation and interactions of fused domains, but they provide a structure for investigating new protein structures that do not exist in nature. Relational algebra is suggested as a potent technique for detecting functionally connected proteins in domain fusion analysis. This approach utilizes extensive domain databases like Pfam and InterPro to anticipate domain fusions and their potential functional associations [165]. Furthermore, the design of inter-domain linkers plays a vital role in preserving the structural integrity and functionality of fused proteins. Recent investigations have identified the ideal features of linkers that prevent undesirable interactions and improve protein stability [166]. Deep learning techniques, like those used in DeepAssembly, enhance the prediction of multi-domain protein structures by properly simulating inter-domain interactions and boosting the accuracy of domain assembly [167]. These computational breakthroughs not only make it easier to design proteins with improved functions, but also broaden the range of possible uses for modified proteins in areas like drug discovery and synthetic biology (Figure 5E).

#### 6.4.2. Rational Design of Chimeric Proteins

Computational techniques have greatly advanced the rational design of chimeric proteins, which entails strategically fusing different protein domains to form multifunctional proteins. These methods utilize knowledge about the structure and function of proteins to direct the merging of protein domains, with the goal of improving or introducing new functions. For instance, the utilization of computational tools such as Protlego simplifies the process of designing and analyzing chimeric proteins by automating the selection and combining of protein fragments. This is accomplished by considering evolutionary conservation and structural compatibility [168]. This strategy has been confirmed by effective applications in producing proteins with enhanced stability and catalytic capabilities, as shown in studies that focus on chimeric enzymes combining domains to boost biocatalytic efficiency [169]. In addition, the combination of machine learning and structural databases, including the Protein Data Bank, enables precise forecasting of domain interfaces and the enhancement of linker regions. These regions are essential for preserving the structural integrity and functionality of the chimeras [164]. These developments not only simplify the design process but also broaden the possible uses of chimeric proteins in the creation of therapies, synthetic biology, and industrial biotechnology. With the ongoing advancement of computational tools, there is a potential for significant improvement in the accuracy and effectiveness of chimeric protein design. This progress opens up opportunities for groundbreaking solutions in diverse scientific disciplines (Figure 5E).

## 7. Case Studies and Applications in Biotechnology and Pharmaceuticals

### 7.1. Engineered Antibodies and Immunotherapeutics

#### 7.1.1. Computational Design of Antibody–Antigen Interfaces

**Figure 6:**
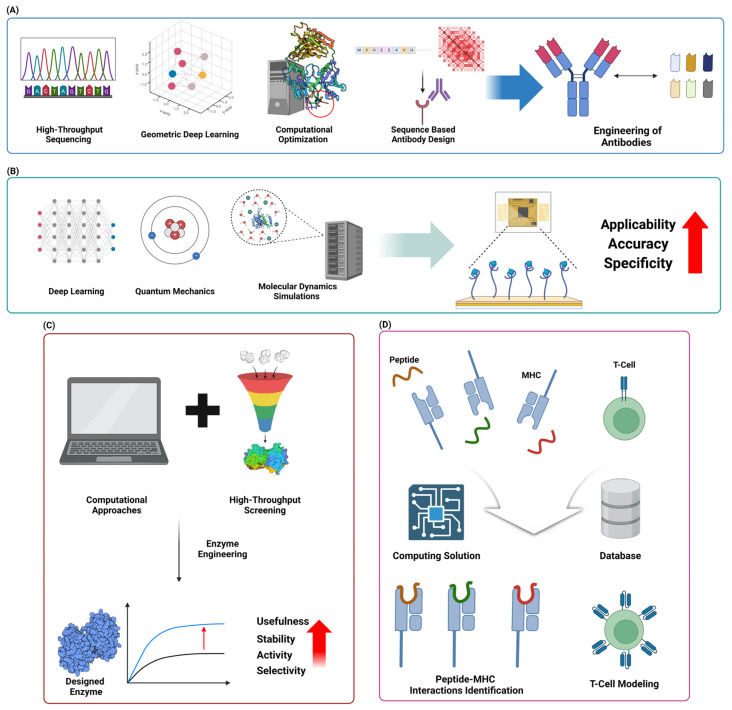
Protein engineering applications using computational approaches in biotechnology and pharmaceuticals. (**A**) High-throughput sequencing data and geometric deep learning can enhance antibody binding prediction capabilities. Computational technologies such as deep learning enable sequence-based antibody design, providing advanced approaches to antibody engineering. (**B**) Computational and structural methods, such as deep learning and quantum mechanical molecular dynamics simulations, have enabled the prediction of atomic-level movements of biomolecules, leading to improvements in the applicability, accuracy, and specificity of protein-based biosensors. (**C**) Advancements in computational technologies such as machine learning, combined with high-throughput screening, have enabled improved enzyme engineering with enhanced catalytic properties, leading to increased stability, activity, and selectivity of enzymes. (**D**) Computational technologies play a crucial role in therapeutic protein design, particularly in predicting peptide-MHC binding affinity. These methods not only advance personalized medicine but also accelerate the clinical application of protein therapeutics.

The use of advanced algorithms in computational design has greatly improved the production of modified antibodies and immuno-therapeutics by enhancing the prediction and optimization of binding interactions in antibody–antigen interfaces. The utilization of computational approaches, as exemplified by Norman et al., involves the use of structural modeling to discover crucial residues in antibody-antigen interactions. This process aids in the development of antibodies with enhanced specificity and affinity [170]. Machine learning techniques, such as Parapred, which is a deep learning algorithm, have been used to forecast paratope areas. This has resulted in enhanced precision in antibody design by specifically targeting important binding sites [78]. By combining computational methodologies with high-throughput sequencing data, it has been possible to create more potent therapeutic antibodies. This approach allows for the quick evaluation and enhancement of potential antibody candidates [171]. Moreover, the application of geometric deep learning has enhanced the ability to forecast protein interaction surfaces, offering valuable knowledge about the structural factors that influence antibody–antigen binding and assisting in the development of innovative antibody forms [78]. The computational breakthroughs not only simplify the process of designing antibodies but also broaden their potential for use in treating many diseases. This is evident from the growing number of computationally produced antibodies that are being tested in clinical studies [172]. As the field progresses, these methods hold the potential to improve the accuracy and effectiveness of antibody-based treatments, aiding in the advancement of advanced immunotherapies (Figure 6A).

#### 7.1.2. In Silico Optimization of Antibody Stability and Specificity

The latest progress in the computational optimization of antibody stability and specificity has greatly improved the creation of engineered antibodies and immunotherapeutics. This is achieved by using computational approaches to simplify and increase the process of designing antibodies. The computational approach, as outlined by Norman et al., employs structural modeling to forecast and improve the stability and specificity of antibodies. The main focus is on optimizing specific residues at the interface between the antibody and antigen to enhance binding strength and decrease the likelihood of immune response [170]. Deep learning algorithms, such as DeepAb, have been utilized to directly forecast the structures of antibody Fv based on their sequences. This allows for the creation of improved variants with higher thermostability and affinity, eliminating the requirement for considerable experimental data [173]. These models combine high-throughput sequencing data and machine learning to quickly evaluate and improve antibody candidates, resulting in a significant reduction in the time and cost required by traditional experimental methods [171]. In addition, the incorporation of artificial intelligence in the process of creating antibodies has made it possible to anticipate the specificity of antigens based on antibody sequences. This has enabled the production of synthetic antibodies that have enhanced binding properties [171]. As these computational techniques advance, they provide the potential to improve the accuracy and effectiveness of antibody optimization. This progress will facilitate the creation of next-generation immunotherapeutics with enhanced therapeutic characteristics (Figure 6A).

### 7.2. Biosensors and Diagnostics

#### 7.2.1. Rational Design of Protein-Based Biosensors

The latest progress in the logical development of protein-based biosensors has greatly improved their use in biotechnology and diagnostics. This has been achieved by utilizing computational and structural methods to boost the binding specificity and sensitivity. Computational techniques, as described by Kaczmarski et al., employ knowledge about the structure and evolution of biosensors to design sensors that have enhanced ability to bind to specific molecules and exhibit improved fluorescence properties. This allows for accurate identification of small molecules in complicated biological settings [174]. The study published in *Nature* showcases the potential of de novo designed protein switches in the development of modular and tunable biosensor platforms. These protein switches can sense a wide range of targets by linking conformational changes to sensitive outputs, thereby enhancing the versatility of biosensor applications [175]. Moreover, the incorporation of synthetic biology methods has enabled the development of genetically engineered biosensors that can actively control metabolic pathways, providing the ability to monitor and manipulate cellular processes in real-time. This has been demonstrated in research involving biosensors based on transcription factors [176]. These improvements enhance the functionality and adaptability of protein-based biosensors, making them suitable for various applications like environmental monitoring, healthcare diagnostics, and industrial biotechnology. The advancement of computational tools and synthetic biology is anticipated to boost the precision and efficiency of protein-based biosensors, facilitating the development of creative solutions for intricate analytical problems.

#### 7.2.2. Computational Approaches for Enhancing Sensor Sensitivity and Specificity

Advancements in computational techniques have greatly enhanced the sensitivity and specificity of biosensors, leading to their increased use in the biotechnology and pharmaceutical industries. The enhancements are primarily propelled by the incorporation of sophisticated algorithms and simulations that enhance the efficiency of sensor functionality. The use of molecular dynamics simulations and quantum mechanics computations has played a crucial role in accurately predicting the behavior of biomolecules at the atomic level. This enables the precise adjustment of biosensor components to achieve certain performance characteristics [177,178]. Computational fluid dynamics has been used to improve the advancement of microfluidic devices, which are important for enhancing the sensitivity and specificity of biosensors by regulating fluid dynamics and analyte transport. In addition, researchers have used hybrid computational methods that combine molecular docking and virtual screening to discover new sensing components that have both high specificity and affinity. This has enabled the creation of biosensors that can detect low levels of target substances in complex biological samples [179]. Machine learning and artificial intelligence have improved biosensor design, providing new opportunities to enhance the predictive capability and precision of computational models, hence facilitating the creation of more advanced biosensing devices [178]. As these computational tools progress, they hold the potential to enhance the field of biosensors, making them more efficient for use in healthcare diagnostics, environmental monitoring, and food safety (Figure 6B).

### 7.3. Industrial Enzymes

#### 7.3.1. Computational Engineering of Enzymes for Biocatalysis

Computational engineering of industrial enzymes for biocatalysis is an advanced field in biotechnology and pharmaceuticals that aims to improve enzyme functioning for industrial use. Improvements in machine learning have had a substantial impact on enzyme engineering. These improvements provide tools to predict interactions between enzymes and substrates, which is essential for designing enzymes with improved catalytic characteristics [180]. By combining computational approaches with high-throughput screening, researchers may effectively explore large enzyme design spaces. This enables the synthesis of stable and selective biocatalysts that are essential for cost-effective bio-based processes [87]. In addition, the combination of molecular dynamics simulations and ML models allows for a detailed understanding of enzyme processes at the atomic level. This enables precise adjustments that improve enzyme stability and activity in industrial settings. The combination of computational and experimental methods has resulted in the successful modification of enzymes to perform new tasks, increasing their usefulness in drug production and environmental cleanup [181]. These advancements highlight the significant impact of using computational enzyme engineering to develop environmentally-friendly and effective biocatalytic processes. This, in turn, enhances the capacities of the biotechnology and pharmaceutical industries (Figure 6C).

#### 7.3.2. Design of Enzymes for Biodegradation and Environmental Applications

Enzyme design for biodegradation and environmental applications is a rapidly growing area in biotechnology, propelled by breakthroughs in protein engineering and computational techniques. A recent study emphasizes the utilization of directed evolution and rational design to augment the enzymatic capacity to break down persistent pollutants, including plastics and other synthetic substances, aiding in environmental preservation [182]. Enzymes that have been specifically designed have been enhanced to break down polyethylene terephthalate (PET), a commonly used plastic. This has been achieved by improving their ability to speed up chemical reactions and their ability to remain stable over time. This demonstrates the promise of using biological catalysts in recycling and managing garbage [183]. In addition, the combination of computational modeling and experimental methods has made it possible to create enzymes that can work under harsh environmental circumstances, thereby expanding their usefulness in various industrial processes [184]. These advancements highlight the significant impact of enzyme engineering in tackling environmental issues, providing sustainable methods for managing pollutants and recovering resources (Figure 6C).

### 7.4. Therapeutic Protein Design

#### 7.4.1. Computational Approaches for Improving Protein Drug Properties

The field of therapeutic protein design has experienced notable progress, especially with the incorporation of computational methods that improve the feasibility of developing protein-based therapeutics. Computational methods, such as molecular dynamics and artificial intelligence, play a crucial role in tackling important aspects of therapeutic proteins, such as affinity, selectivity, stability, and solubility. These factors are essential for the successful application of these proteins in clinical settings [185]. These techniques allow for the anticipation and enhancement of protein structures, making it easier to create proteins with enhanced therapeutic characteristics. For example, deep learning algorithms have been used to forecast protein interactions and improve sequences to decrease immunogenicity and increase stability. These computational solutions not only make the medication development process more efficient but also save expenses by reducing the necessity for large experimental trials [6]. The collaboration between computational scientists and pharmaceutical developers is essential for closing the divide between theoretical models and real applications, guaranteeing the appropriate utilization of computational tools in drug discovery [185]. As these technologies continue to advance, they hold the potential to greatly transform the process of designing therapeutic proteins. They offer more accurate and effective methods for building new protein-based therapeutics [147] (Figure 6D).

#### 7.4.2. In Silico Prediction of Immunogenicity and Optimization of Protein Therapeutics

The topic of in silico prediction of immunogenicity and optimization of protein therapeutics is fast advancing, utilizing computational technologies to improve the safety and effectiveness of biologic medications. These methods are crucial for detecting possible immune-stimulating regions in protein-based treatments, enabling their alteration or removal prior to use in clinical settings. Machine learning algorithms have been recently combined with classical bioinformatics methods to identify T-cell epitopes. This is accomplished by analyzing peptide–MHC binding affinities, which is important for evaluating immunogenic potential [186,187]. The utilization of extensive databases such as the Immune Epitope Database (IEDB) has enabled the refinement of these algorithms, enhancing their precision and suitability across various HLA haplotypes [186]. In addition, computational techniques are used to enhance protein sequences by minimizing their immunogenicity while ensuring their therapeutic effectiveness. This approach tackles obstacles such as MHC polymorphism and the intricate nature of peptide–MHC interactions [186,187]. Recent advances in deep learning have improved T-cell receptor (TCR) modeling and design. The TCRmodel2, created by Yin et al., advances deep learning-based high-resolution TCR recognition modeling [188]. AlphaFold is adapted to model TCR–peptide–MHC complexes from sequence data, improving accuracy over earlier methods. Sidhom et al.’s DeepTCR framework used deep learning to reveal TCR sequence-based characteristics [189]. This combination of unsupervised and supervised learning algorithms learns joint TCR representations from CDR3 sequences and V/D/J gene use to model complex TCR sequencing data. Ribeiro-Filho et al. compared ProteinMPNN and ESM-IF to standard physics-based TCR design methods to investigate structure-based deep learning algorithms. These methods may help create fixed-backbone TCRs that bind MHC-presented target antigenic peptides. Katayama et al. also reviewed machine learning approaches to TCR repertoire analysis, noting the growing use of deep learning for antigen specificity prediction and TCR sequence synthesis [190]. TCR modeling and design is evolving rapidly, with new methods like TCR-VALID by Widrich et al. developing capacity-controlled disentangling variational autoencoders for meaningful continuous representations of TCR sequences [191]. In silico methodologies not only optimize the drug development process by minimizing the requirement for extensive in vitro and in vivo testing but also facilitate the tailoring of protein treatments to unique patient profiles, hence boosting personalized medicine [187]. As these technologies progress, they have the potential to greatly decrease the failure rates of protein therapies due to immunogenicity, therefore speeding up their journey towards clinical application [187] (Figure 6D).

## 8. Challenges and Future Perspectives

### 8.1. Integration of Multi-Scale Modeling Approaches

**Figure 7:**
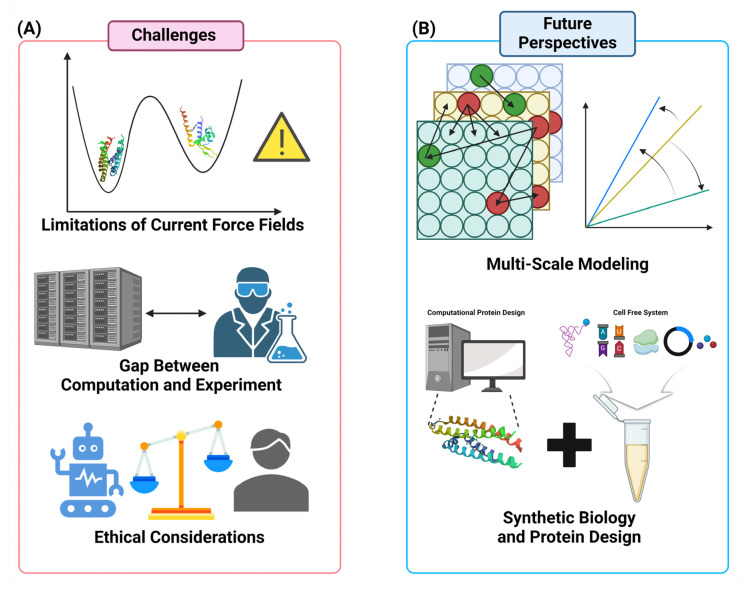
Challenges and future perspectives in computational approaches to protein engineering applications. (**A**) Current force fields have limitations in accurately capturing changes in electrostatic interactions, which impacts the accuracy and reliability of simulations. Integrating computational tools with experimental validation is essential for enhancing the accuracy and efficiency of protein design. Ethical issues related to bias, transparency, and accountability arise in the application of AI in protein engineering. (**B**) The integration of multi-scale modeling approaches is essential for understanding the complex dynamics of protein systems and developing proteins with new functions, and the advancement of these models holds great potential in the field of computational protein design. The combination of computational protein design and synthetic biology enables the development of innovative proteins.

The incorporation of multi-scale modeling methods in computational protein engineering poses obstacles and offers future prospects for enhancing molecular design. Multi-scale modeling is crucial for understanding the intricate dynamics of protein systems at several levels, ranging from electronic to macroscopic, by integrating atomistic, coarse-grained, and continuum models. This methodology overcomes the constraints of conventional methods that face difficulties in dealing with the extensive range of protein conformations and the lengthy simulation times needed for in-depth protein investigations [192]. Machine learning has recently made significant progress in enhancing multi-scale modeling. This progress has resulted in improved prediction accuracy and the ability to efficiently explore protein design spaces [193]. These computational tools aid in the discovery of protein structures and interactions, which are essential for the development of proteins with new activities and enhanced stability. Nevertheless, there are still obstacles to overcome when it comes to merging data from various scales and guaranteeing that models precisely depict biological phenomena. Future prospects involve the creation of hybrid models that effortlessly combine different scales, aided by advancements in processing power and algorithms [6]. As these models advance in complexity, they have the capacity to transform protein engineering by offering comprehensive understanding of protein activity, thereby expediting the creation of new medicines and biomaterials (Figure 7B).

### 8.2. Addressing the Limitations of Current Force Fields

Overcoming the constraints of existing force fields in computational protein engineering and molecular design is a crucial task that greatly affects the precision and dependability of molecular simulations. Conventional force fields commonly utilize stationary charges located at the atoms, which may not accurately capture the changing behavior of electrostatic interactions. As a result, this can lead to mistakes when simulating protein folding and interactions [194,195]. Polarizable force fields, such as the Drude and AMOEBA models, have been developed to incorporate electronic polarization effects. These improvements aim to enhance the accuracy of representing molecular interactions and energy landscapes [194,196]. Nevertheless, these models require significant computational resources and can be very responsive to initial conditions, which presents obstacles to their extensive implementation [195,196]. Integrating both polarizable and non-polarizable elements in hybrid models is a potential strategy to achieve a compromise between accuracy and computational efficiency [194,195]. Furthermore, the application of machine learning and automated fitting techniques has demonstrated promise in improving force field parameters by utilizing extensive datasets of experimental and simulation data [194]. The increasing computer capacity allows for the integration of advanced force fields with multi-scale modeling techniques. This integration is expected to improve the accuracy of simulations, making it easier to design proteins with new functionalities and better stability [185] (Figure 7A).

### 8.3. Bridging the Gap between Computation and Experiment

The integration of modern computational tools with empirical validation is crucial for bridging the gap between computational and experimental approaches in protein engineering and molecular design. This integration aims to enhance the design and functionality of proteins. Advancements in computational technologies, including machine learning and artificial intelligence, have greatly enhanced the accuracy of predicting protein structures and identifying functional areas. This has made it easier to tailor protein functionalities with more precision [3,197]. Nevertheless, due to the intricate nature of biological systems and the constraints of computer models, it is essential to conduct experimental verification in order to guarantee the dependability of these forecasts [198]. The emergence of platforms such as Mutexa showcases the endeavor to establish intelligent protein engineering ecosystems that integrate high-throughput computation with bioinformatics and quantum chemistry. This integration aims to simplify the process of identifying potential protein variants that show promise [4]. Furthermore, the combination of computational and experimental methods might expedite the design process by enabling the development of targeted libraries for laboratory evolution, thus minimizing the extensive sequence space that requires sampling [199]. With increasing computer power and advancement of algorithms, the combination of computation and experimentation has the potential to greatly impact protein engineering. This could result in the creation of new proteins that have improved stability, activity, and therapeutic properties [147] (Figure 7A).

### 8.4. Ethical Considerations in AI-Driven Protein Engineering

The incorporation of AI into protein engineering and molecular design gives rise to noteworthy ethical concerns that want attention in order to guarantee responsible and advantageous progress in the domain. The utilization of AI in protein engineering has significant promise for the creation of innovative medicines and biomaterials. However, it also presents concerns of bias, transparency, and accountability. The main ethical concerns with AI systems are centered around their ability to perpetuate pre-existing biases present in the training data, resulting in unfair outcomes in healthcare applications [200,201]. Furthermore, the capacity to provide clear explanations for AI models is essential in order to uphold trust and guarantee that AI-driven decisions in protein design are visible and comprehensible to stakeholders [202]. Researchers and developers are encouraged to actively participate in ethical frameworks and principles that prioritize fairness, the prevention of harm, and the respect for human autonomy in the implementation of AI applications [201,203]. Additionally, it is imperative for scientists, ethicists, and legislators to work together in order to establish strong governance systems that effectively tackle ethical dilemmas and encourage the conscientious application of AI in protein engineering. In order to maintain a balance between innovation and societal values and to prevent the misuse of AI technology, it is crucial for the field to engage in ongoing debate and adjust ethical standards as it evolves [204] (Figure 7A).

### 8.5. Emerging Opportunities in Synthetic Biology and Protein Design

The integration of modern computational tools is driving emerging opportunities in synthetic biology and protein design, which have transformational potential in the fields of biotechnology and molecular design. Synthetic biology, a field that focuses on creating new biological components and systems, is using machine learning more and more to improve protein engineering. This allows for the development of proteins with new functions and better performance in industrial and medical applications [205]. Cell-free protein synthesis (CFPS) is a promising technique that enables the quick prototyping and manufacturing of proteins without the limitations of living cells. This method facilitates the investigation of novel protein designs and functionalities [206]. Moreover, the merging of synthetic biology and metagenomics is creating opportunities to construct intricate biological systems, hence improving our capacity to control and exploit microbial populations for biotechnological purposes [207]. However, there are still difficulties in expanding the use of these technologies and making sure that they are available to a wider group of academics. This is crucial in order to fully utilize their potential in addressing global issues like sustainable development and healthcare. [197,208]. The advancement of computational tools and their integration with experimental methodologies is paving the way for groundbreaking innovation and application of protein design in synthetic biology across several domains (Figure 7B).

## 9. Conclusions

The domain of computational protein engineering and molecular design is swiftly progressing, propelled by improvements in machine learning, molecular modeling techniques, and high-performance computing. This study has emphasized the wide range of applications and creative methods in this rapidly evolving subject, including AI-powered protein design, molecular dynamics research, and computational drug discovery. In the future, it will be essential to combine these computational methods with experimental validation in order to fully realize their promise. The ongoing advancement of increasingly precise and effective algorithms, together with the growing accessibility of biological data, holds the potential to expedite the identification and creation of new proteins and molecules with improved capabilities. The research showcased in this Special Issue of *Molecules* highlights the significant influence of computational methods on protein engineering and molecular design. As these methodologies progress and develop further, they will surely have a growing impact on our comprehension of biological systems and the creation of inventive solutions to urgent difficulties in biotechnology, medicine, and other fields.
